# Some Spinal Cord Lesions

**Published:** 1911-09-23

**Authors:** 


### Neurology.

### SOME SPINAL CORD LESIONS.

### VII.—HEREDITARY ATAXY (FRIEDREICH'S DISEASE).

This is a chronic disease of the spinal cord, which is characterised by ataxy, and later by weakness in the legs. It occurs as a rule in several members of the same family.

The lesion is a combined sclerosis of the posterior and lateral columns of the cord. The posterior columns are most extensively affected in the lumbar region, the posterior-median, posterior-external columns, and posterior root-zones being involved. In the dorsal and cervical regions all these parts may be equally affected, or the posterior root-zones and posterior median columns only. The posterior nerve-roots usually show degenerative changes as in locomotor ataxy. The pyramidal tracts, both crossed and direct, are sclerosed throughout the cord, and the adjacent direct cerebellar tract may also be involved. The sclerosis in this disease is more extensive than in ataxic paraplegia, the posterior root-zones being involved in all the regions of the cord. It differs from locomotor ataxy in having the pyramidal tracts and cerebellar fibres sclerosed as well as the posterior columns and posterior root-zones. The ataxy and loss of knee-jerk are explained by the sclerosis of the posterior columns, including the posterior root-zones, and the weakness and stiffness of the limbs by the sclerosis of the pyramidal tracts.

Males are slightly more liable to this disease than females. The first symptoms may be recognised between the ages of 2 and 24, most frequently between 7 and 8 and 12 and 16. In isolated cases the onset is sometimes later.

Unsteadiness in walking and standing, a tendency to stumble and fall, and progressive weakness of the legs are the earliest indications of the disease. This inco-ordination gradually increases until the patient can only stand with the feet wide apart. Inco-ordination of the arms also occurs, but not as a rule until after the legs have become almost paralysed. When the disease has fully developed, the following symptoms may be present.

There may be considerable loss of power in the legs, the flexor muscles being more affected than the extensors. The gait is irregular and swaying in character, resembling that of a drunken man. The heels are not forcibly brought to the ground as in locomotor ataxy. Romberg's sign may or may not be present. The movements of the arms and head are irregularly jerky in character. As a result of the weakness of the flexors of the ankles, talipes equinus usually occurs, and lateral curvature of the spine from weakness of the muscles of the back. The big toe is generally dorsally flexed on the first phalanx—a kind of permanent extensor plantar reflex—and there is well-marked pes cavus.

As a rule there is no loss of sensation. In some cases slight paræsthesia may be present in patches. There are neither lightning pains nor visceral crises. The knee-jerks are absent, and there is no ankle clonus, but the plantar reflexes are extensor in character. There is no wasting of the muscles until quite late in the disease, though they may seem to be atrophic, owing to non-development.

The electrical reactions are usually normal. The functions of the bladder and rectum are not as a rule affected. Sexual power is generally absent.

Slight but definite nystagmus is present in most cases. The pupils react both to light and to accommodation. There is neither oculo-motor paralysis nor optic atrophy. Mental changes are absent. The speech may be slow and scanning in character.

The progress of the disease is slow in the majority of cases, but surely progressive towards a fatal termination, which in some cases may not occur for more than thirty years. The chief diagnostic signs are: the early onset, the occurrence of the disease in several members of the same family, the characteristic inco-ordination, the nystagmus, the loss of knee-jerks associated with extensor plantar reflexes, the scanning speech, and, lastly, but by no means the least important, the lateral curvature of the spine, the hallux erectus, and the pes cavus.

